# Is anemia a probable cause of fatigue in patients with multiple sclerosis?

**Published:** 2013

**Authors:** Soha Mir-Reza, Maryam Tabatabaeiyan, Rozita Doosti, Mahsa Owji, Abdorreza Naser Moghadasi

**Affiliations:** Researcher, Neuroscience Institute, Iranian Center of Neurological Research, Sina Multiple Sclerosis Research Center, Tehran University of Medical Sciences, Tehran, Iran

**Keywords:** Anemia, Fatigue, Multiple Sclerosis

Fatigue, in many studies, has been identified as the most common complaint of patients with Multiple Sclerosis (MS); it can have a negative impact on different aspects of their life.^1^ Fatigue in MS is a complex process which can occur due to factors directly related to the disease or other secondary complications.^1^ Of the primary factors, hypometabolism in the frontal cortex and basal ganglia, and hypothalamic-pituitary-adrenal axis and axonal damage can be noted. Of the secondary factors, depression and sleep disorders should be mentioned.^1^

Until now, anemia has not been studied as the cause of fatigue in MS patients.

In our study, we evaluated the relationship between anemia and fatigue in patients with MS in order to see if anemia could be considered as a potential cause of fatigue in patients with MS.

This study was performed at Sina MS Research Center, Iran, and all steps were approved by the local Ethics Committee at Tehran University of Medical Sciences.

43 patients were enrolled, and the diagnosis was made according to the McDonald criteria. There were no limitations of age, sex, duration, type of disease, and type of medication. The patients did not use iron or vitamin B12, or folic acid-containing drugs.

5 ml blood samples were collected from all patients admitted to Sina MS Research Center for complete blood count (CBC) test and the encoding was sent to the special lab. The measurement method of hemoglobin was high performance liquid chromatography (HPLC).

FSS (Fatigue Severity Scale) questionnaire was used to evaluate fatigue. 31 female patients and 12 male patients were included in this study. The participants aged between 17 to 56 years with the average age of 30 ± 8.2. The average rate fatigue in these patients was 4.35 based on the FSS questionnaire (4.53 in men and 4.32 in women).

The diagnosis of anemia was based on hemoglobin of below 12.5 g/dl in women and 13.5 g/dl in men.^2^ As a result, based on these criteria, 8 patients were anemic and 35 were non-anemic. The average hemoglobin in anemic and non-anemic groups was 12.38 g/dl and 13.90 g/dl, respectively. The average of mean corpuscular volume (MCV) in anemic and non-anemic groups was 86.87 fL and 87.14 fL. The rate of fatigue in anemic and non-anemic patients was 4.44 and 4.34, respectively. They had no statistically significant difference (P = 0.055).

Our study shows that anemia has no role in creating fatigue in MS patients. The authors are aware that the study sample was not large enough; the inclusion criteria (including lack of drugs with folic acid combination, iron and vitamin B12) restricted the number of patients participating in the study.

Anemia is a common disorder in the world. No studies have been performed on the comparison of fatigue among non-MS anemic patients and MS patients. However, descriptions of fatigue in MS patients are completely unique and MS patients say that they had not experienced the same fatigue previously.^3^ MS-related fatigue, causing severe disruption in normal daily activities, is fundamentally different from the fatigue that healthy people experience.^4^ This fact suggests that fatigue in MS patients is due to MS pathology. This is also confirmed in other studies.

As noted, the PET scan showed hypometabolism in the frontal cortex and basal ganglia of MS patients, who also suffer from fatigue at the same time.^1^ On the other hand, it is found that creatine: N-acetylaspartate ratio in the brain of these patients is significantly lower than those who do not suffer from fatigue. It seems that decrease in creatine:N-acetylaspartate ratio in the brain is because of axonal loss in MS pathophisiology.^1^ These findings suggest that at least a part of the pathophysiology of fatigue is central.

Although secondary causes of fatigue in MS patients, including sleep disorders and depression have been discussed, the unique feature of fatigue in MS patients is due to the pathophysiology of MS itself and not the secondary causes such as anemia.^1^
